# Assessment of Genetic Diversity in *Bupleurum* spp. Basing Agronomic Traits, Medicinal Components and ISSR Markers

**DOI:** 10.3390/genes14040951

**Published:** 2023-04-21

**Authors:** Yiqing Peng, Alam Nafee-Ul, Mingzhi Liu, Qiuling He, Zongsuo Liang

**Affiliations:** 1 College of Life Sciences, Northwest A & F University, Xianyang 712100, China; pyq1@nwafu.edu.cn; 2 College of Life Sciences and Medicine, Zhejiang Sci-Tech University, Hangzhou 310018, China

**Keywords:** *Radix bupleuri*, agronomic traits, HPLC, ISSR, genetic diversity

## Abstract

*Radix bupleuri* is one of the bulk medicinal materials in China and it is widely adopted in clinical applications and drug discovery. The investigation of agronomic traits, active component content and genetic diversity in diverse *Radix bupleuri* germplasms may provide evidence to promote the selection of better strains. In this research, 13 germplasms from various sources were used to investigate the variations between different *Radix bupleuri* germplasms. Nine biological characteristics were noted in the field, and the levels of the two primary active ingredients were determined using high performance liquid chromatography (HPLC). Moreover, the molecular marker technique of inter-simple sequence repeat (ISSR) and the unweighted pair group method with arithmetic means (UPGMA) were employed to evaluate the molecular genetic diversity. The findings showed that there was a wide range of variation among the many varieties of *Radix bupleuri*, with coefficients of variation for agronomic traits and active component content ranging from 7.62% to 41.54% and 36.47% to 53.70%, respectively. Moreover, there are different degrees of relationship between the two. Since there was a significant correlation between root weight and saikosaponin content, it was possible to classify a plant based on its weight and anticipate its saikosaponin content. The 13 species were divided into four groups based on their germplasm by genetic markers-based cluster analysis. This indicated the possibility that the component content would not necessarily be related to germplasm and might easily be influenced by environmental factors. The use of ISSR marker technology made it possible to precisely identify the various *Radix bupleuri* provenances and its counterfeit products. There may be a way to prevent the misunderstandings caused by the appearance and composition of Chinese medicinal substances. In our study, the germplasm of *Radix bupleuri* that was widely circulated in the market was comprehensively evaluated in terms of agronomic traits, active components and molecular level, and identified by simple means, to provide a theoretical basis for the evaluation and screening of fine germplasms of *Radix bupleuri*.

## 1. Introduction

*Radix bupleuri*, a perennial herb, is also known as mushroom grass, firewood grass, and so on. *Radix bupleuri* dried root, which originates from *Bupleurum Chinense* DC. and *Bupleurum scorzonerifolium* Willd. of the genus *Bupleurum* (*Bupleurum* L.) in the Umbelliferae family (Umbelliferae), is commonly used as a medicine and has a bitter and pungent taste. In China, *Radix bupleuri* is extensively present in Shaanxi, Shanxi, Gansu, Hebei, Inner Mongolia and other regions [1]. As a bulk medicinal material in China, *Radix bupleuri* has the effects of harmonizing the exterior and interior, soothing the liver, relieving depression, and raising yang energy [2]. It also plays a role in treating exogenous fever, stagnation of liver-qi, hypochondriac pain and chest distress [3]. Saikosaponin is the main effective medicinal component of *Radix bupleuri*, with anti-inflammatory [4], anti-cancer [5], anti-depressant [6], antioxidant [7] and anti-liver-fibrosis effectiveness [8]. Recent medical research has exhibited that all 23 main active ingredients from *Radix bupleuri-Paeonia lactiflora* compound medicine acted on potential depression target proteins and alleviated depressive symptoms [9]. A compound of *Radix bupleuri* and *Radix Scutellariae* called ‘Chaiqin Kanggan Mixture’ had a substantial impact in preventing coronavirus disease 2019 (COVID-19) and influenza virus infection [10]. The market demand for *Radix bupleuri* is currently steadily rising as more and more pharmaceutical research findings are transitioning to industrialization. Expanding the breeding of high-quality *Radix bupleuri* to increase yield for production will be the future trend.

However, high-quality *Radix bupleuri*, or even the correct provenance selection of *Radix bupleuri*, often causes confusion for growers. In addition to the above two kinds of *Radix bupleuri* (*B. chinense* DC. and *B. scorzonerifolium* Willd.) stipulated in the Pharmacopoeia of the People’s Republic of China (2020 version) [11], there are many species of *Radix bupleuri* sold in the domestic medicinal material markets. As many as 44 species, 17 varieties and seven forms of *Radix bupleuri* plants are used as medicinal herbs [12]. They are used medicinally differently in various regions. In Guizhou Province, the medicinal standard contained *Bupleurum marginatum* var. *stenophyllum* (Wolff) Shan et Y. Li. and *Bupleurum scorzonerifolium* Willd [13]. *Bupleurum smithii* Wolff were officinal herbs in Gansu Province [14]. In the early 1970s, *Bupleurum falcatum*, a medicinal plant originally came from Japan, was introduced into China and cultivated in large quantities in Shandong, Hebei and other places, and mostly sold to Japan and Korea [15]. Although the national and local standards positively regulated the medicinal species of *Radix bupleuri*, counterfeits and substitutes are still available on the market. Many studies have demonstrated that the morphology, component composition and other characteristics of various *Radix bupleuri* species vary greatly [16]. However, growers who only rely on experience to judge the quality of varieties and their quality according to the appearance of plants often make mistakes, which further affects the efficacy and stability of downstream products. Therefore, it is necessary to explore a reliable and simple means of germplasm identification.

The species diversity of medicinal plants used as root medicines is mainly reflected in their morphological variations in the aboveground part, the content of active components in the underground part, and the molecular level. At present, the investigation of germplasm resources of *Radix bupleuri* is from a single point of view, only considering the characteristics of one aspect. Zhang et al. compared several agronomic traits of various *Radix bupleuri* species and found that the leaf characters of different types varied considerably and were significantly correlated with yield [17]. Li et al. identified the main active ingredients of *Radix bupleuri* from nine distinct sources and showed that the variation coefficient of the total content of saikosaponin a (SSa) and saikosaponin d (SSd) was 38.9%, and the difference between various *Radix bupleuri* was significant [18]. Wei et al. applied simple repeat sequence (SSR) molecular marker technology to identify six varieties of *Radix bupleuri* and reported promising results for different species identification but unsuitable identification of similar species [19]. Simple repeat amplification (ISSR), a molecular marker method based on microsatellite sequence, has established itself in recent years. Compared with random amplified polymorphic DNA (RAPD) and amplified fragment length polymorphism (AFLP), ISSR has the advantages of strong polymorphism, high stability, reliable repeatability, cheap cost and straightforward operation [20,21]. A reliable ISSR-PCR reaction system for *Radix bupleuri* has been developed [22]. The genetic relationship of 11 *Radix bupleuri* was identified and analyzed, and 156 polymorphic bands were obtained, which accounted for 84.3% of all bands [23]. Therefore, ISSR technology may have favorable effects in identifying *Radix bupleuri* species, molecular marker-assisted selection breeding and genetic diversity research.

In this study, the species diversity of 13 different *Radix bupleuri* germplasms from the Baoji base in Shaanxi Province was investigated based on their phenotypic characteristics, saikosaponin content and molecular marker profiles during the growth periods. The germplasm difference of *Radix bupleuri* was explored from multiple angles, and a simple and convenient germplasm identification method was explored and aimed to offer a theoretical and practical foundation for breeding new cultivars of *Radix bupleuri*, germplasm innovation, and effective utilization of medicinal resources in China.

## 2. Materials and Methods

### 2.1. Plant Material

*Radix bupleuri* germplasm resources were provided by Boren Pharmaceutical Co. Ltd., Baoji City, Shaanxi Province (Table 1), and planted in the field of Chencang District (106.5437° E, 34.4645° N, 553 m above sea level), Baoji City, Shaanxi Province in 2018. The full bloom period was 8 August 2019, and the picking period was 7 October 2019. Baoji is a temperate semi-humid climate with a mild climate and abundant rainfall, with an annual average temperature of 13 °C. The annual precipitation is 710–1000 mm, and the precipitation is concentrated in July, August and September, accounting for about 50% of the annual precipitation. This area is suitable for the growth of *Radix bupleuri*.

Five agronomic traits in the aboveground part of *Radix bupleuri* were investigated and analyzed at the full bloom stage (Table 2). According to the number of experimental materials, the trials on these selected strains were conducted using a Complete Randomized Block design (CRBD), a total of 10 plants were labeled, and the labeled plants were measured according to the agronomic shape measurement standard in Table 2. At the same time, the leaves of the labeled plants of *Radix bupleuri* were collected and stored at −80 °C for DNA extraction.

After the maturity of the marked plants, the underground part was excavated, and three agronomic traits were measured and recorded (Table 2). The fresh roots of *Radix bupleuri* were dried at 60 °C, crushed, and passed through an 80 mesh sieve for the determination of SSa and SSd contents. The above plants were identified by Professor Liang Zongsuo of Northwest A & F University as fresh plants of *Radix bupleuri* or other plants of the same genus.

### 2.2. Extraction and Determination of SSa and SSd

The root tissues of 13 germplasms were rinsed with deionized water (Laboratory made) after gathering and quickly placed in an oven (JINGHONG, Shanghai, China) at 50 °C to a constant weight. The dried roots were then ground into powder by grinder (SETM, Zhejiang, China), 0.5 g of which was accurately weighed and immersed in 25 mL extraction solution (ammonia (water):methanol = 5:95 (V)) (Kermel, Tianjin, China) and ultrasonically (SCIENTZ, Zhejiang, China) (Power 200 W, Frequency 40 kHz) extracted at room temperature for 30 h. Next, the extract was filtered, and the filtrate was collected, placed in a 50 °C water bath (keelrein, Shanghai, China) and concentrated at 5 mL. The samples were filtered through a 0.22 μm membrane (Solarbio, Beijing, China) to obtain the sample solution to be finally measured. Standard SSa (CAS:20736-09-8) and SSd (CAS:20874-52-6) (Yuanye, Shanghai, China) were accurately weighed and then dissolved in the extraction solution, to prepare 0.4 mg·mL^−1^ and 0.5 mg·mL^−1^ of standard solution, respectively. The standard solution was added to the appropriate amount of extraction solution to obtain concentrations of 5 μg·mL^−1^, 10 μg·mL^−1^, 15 μg·mL^−1^, 20 μg·mL^−1^ and 25 μg·mL^−1^ standard solution. The sample solution and standard solution were measured by HPLC (Waters, Milford, MA, USA) at 210 nm with a 10 μL injection volume. Retention time and peak area were then recorded. Chromatographic conditions: Column: SunFire C18 (4.6 × 250 mm, 5 μm); Column temperature: 20 °C. The mobile phase was acetonitrile–pure water (Kermel, Tianjin, China; Laboratory made). Gradient elution (0–50 min, 25–90% A. 50–55 min, 90% A. 55–60 min, 90–25% A). Flow rate: 1.0 mL·min^−1^. Three repeats were prepared for each sample.

### 2.3. Isolation of Genomic DNA

Plant tissues were ground under liquid nitrogen to a fine powder (3 samples per species), and total DNA was extracted by plant genomic extraction kits (TIANGEN, Beijing, China). The concentration and purity of DNA were detected by 1% agarose gel electrophoresis and a NanoDrop 2000 (Thermo Scientific, Waltham, MA, USA) [24]. The sample DNA was stored in a refrigerator at −20 °C for further PCR amplification.

### 2.4. ISSR-PCR Amplification of Radix bupleuri

The ISSR primers were designed with reference to the ISSR primer sequences published by Columbia University (New York, NY, USA) (synthesized by Hangzhou Youkang Biotechnology Co., Ltd., Hangzhou, China) [25]. Nine primers with good polymorphism, and precise and reproducible amplification bands were selected (Table 3), and ISSR-PCR was used to amplify 13 DNA samples of *Radix bupleuri*. The reaction system was: 10× PCR buffer 2.5 μL, template DNA 75 ng, MgCl_2_ 1.5 μL (2.5 mmol·L^−1^), primer 2 μL, dNTP 2.5 μL (2.5 mmol·L^−1^), Taq DNA polymerase 0.5 μL (5 U·μL^−1^), ddH_2_O to 25 μL (Vazyme, Nanjing, China). The PCR amplification program was: pre-denaturation at 94 °C for 5 min; 40 cycles of 94 °C for 1 min, 52–58 °C for 1 min (annealing temperature according to the optimal annealing temperature of each primer) and 72 °C extension for 1 min, with a final extension for 10 min at 72 °C [26]. The PCR products were detected by 1.5% agarose gel electrophoresis at 120 V voltage [27].

### 2.5. Statistical Analysis

The evaluation of the data of agronomic features and the content of effective components in the study was carried out using the SPSS 23.0 statistical package program. The Pearson method was used for correlation analysis, and the class average method was used for hierarchical clustering analysis and visualized using R 4.1.3 software. The diversity index (H’) was calculated using the formula: H’=∑(P_i_)(lnP_i_) (P_i_ is the frequency of a trait appearing at level i) [28]. ISSR markers were scored as presence (1) or absence (0) of bands [29]. To evaluate the genetic diversity, DPS 7.5 was used to calculate genetic distance, and the unweighted pair group method with arithmetic means (UPGMA) was used for cluster analysis.

## 3. Results

### 3.1. Agronomic Traits Analysis

The nine main agronomic traits of *Radix bupleuri* from distinct germplasm sources revealed notable differences among various germplasm sources (Table 4). The attributes of the aboveground parts exhibited some variances in the results. Plant height differences between germplasm sources were more pronounced. The highest plant height was 127.00 ± 2.65 cm, which came from DC. in Baoji, while the two cultivars of *B. chinense* DC. from Shanxi had lower plant heights of 102.67 ± 5.51 cm and 104.33 ± 5.13 cm, respectively. The No. 12 strain in Baoji *Bupleuri* had the highest plant stem diameter (4.04 ± 0.29 mm), whereas the No. 8 strain had the smallest (3.19 ± 0.18 mm). There were significant differences in leaf size. No. 1, No. 7 and No. 8 had relatively small leaves with a minimum size of 4.97 ± 0.32 cm^2^, whereas the largest leaves (10.23 ± 2.33 cm^2^) were about twice their size. The characteristics of the underground part were a little bit different. The root of *B. chinense* DC. in Changzhi (No. 3) was slender, but the root weight was significantly higher than other varieties, up to 4.17 ± 0.14 g. In addition, the root weights of No. 11 (2.86 ± 0.73 g) and No. 12 (2.17 ± 0.35 g) were slightly larger. The traits between the remaining strains not mentioned were similar.

Diversity analysis of nine agronomic traits showed that the main morphological characters of different *Radix bupleuri* germplasms had different degrees of variation, with the genotypic coefficient of variation ranging from 7.62% to 41.54% and an average coefficient of variation of 18.62% (Table 5). Among them, the variation coefficient of root weight was the largest, while the coefficient of variation of plant height was the smallest. Except for root weight, the coefficient of variation for all traits was less than 30%. The average coefficient of variation of agronomic traits in the belowground part was 27.46%, which was much higher than that in the aboveground part (14.21%). Among the agronomic traits in the aboveground part, the leaf variation coefficient was greater than that of other traits. It was indicated that the diversity of different *Radix bupleuri* germplasms was mainly reflected in the root characteristics in the belowground part, followed by leaf-related traits. Traits such as plant height and stem diameter were more susceptible to environmental influences and tended to be of the same phenotype. The diversity index was mainly distributed between 1.58 and 1.99, with a mean value of 1.90. Leaf length and leaf width have the highest diversity index, indicating that leaf type was rich in genetic diversity. In addition, the diversity indexes of stem diameter, leaf area, root diameter, and root length were above the mean value, which indicated that the genetic diversity of these traits was also relatively abundant. The diversity index of root weight was lower than 1.7, indicating relatively poor genetic diversity of the traits.

### 3.2. Analysis of Effective Components in Radix bupleuri Germplasms

**Figure 1:**
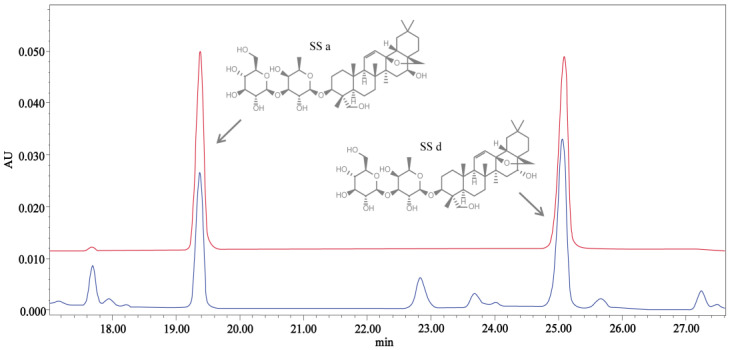
The HPLC chromatogram of two main active components in the *Radix bupleuri* root. The red color chromatogram of SSa and SSd standards, and the blue color chromatogram of *Radix bupleuri*. The chromatographic peaks of the two active components are symmetrical, and the retention time is consistent.

The two saponins in *Radix bupleuri* samples can be completely separated with narrow and symmetrical peaks. The chromatogram is shown in Figure 1, and the determination results are shown in Table 6. The results showed that the content of saikosaponin in 13 *Radix bupleuri* samples was in line with the Chinese Pharmacopoeia (>0.3%). The content of SSd was higher than SSa. The differences in the content of active ingredients in the roots of different varieties of *Radix bupleuri* were highly significant. The highest content of saikosaponin in Changzhi, Shanxi’s *Radix bupleuri* (3.123 ± 0.500%), and saikosaponin in *B. marginatum* var. *stenophyllum* (Wolff) Shan et Y. Li (1.004 ± 0.019% and 0.730 ± 0.053%, respectively,) was significantly lower than other varieties.

The diversity analysis of the saikosaponin content in the roots of different species of *Radix bupleuri* showed that there were different degrees of variation in the content of the two saikosaponins, ranging from 36.29% to 54.18%. The average coefficient of variation value (44.04%) was greater than 30% (Table 7), which showed that the dispersion degree of the content index was large. Compared with the above research results, it was found that the genetic variation of the content of active ingredients was more significant than the morphological traits. However, the diversity indices were mainly distributed from 1.34 to 1.66, with an average of 1.47, all lower than 1.9. It showed that the content of saikosaponin in different varieties of *Radix bupleuri* is quite different, but the content of most varieties tended to be the same, resulting in a decline in the richness of diversity. Some strains possess prominent high-content features that serve as positive references for practical breeding and culture.

### 3.3. Correlation Analysis of Agronomic Traits and Effective Components Content

**Figure 2:**
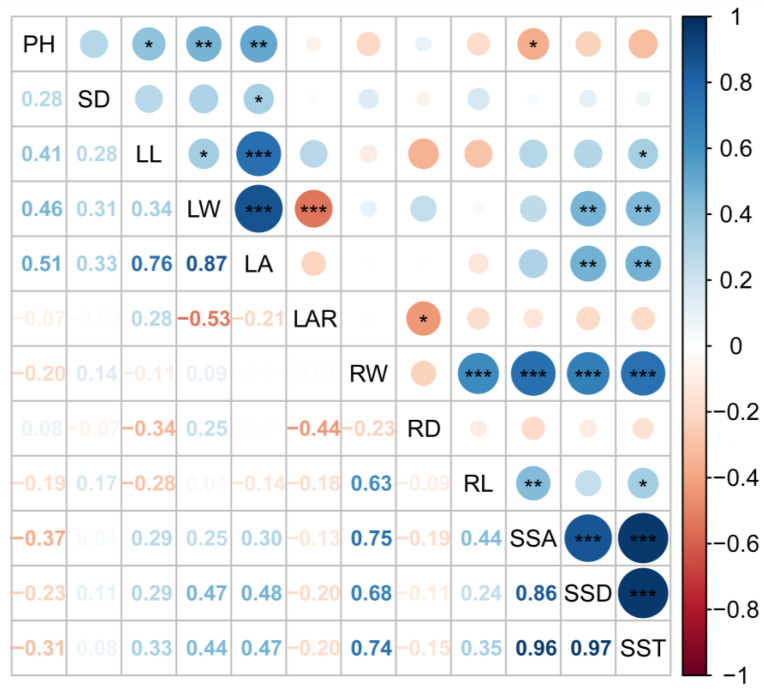
Correlation analysis chart of 12 traits (including major agronomic and content traits) of different germplasm of *Radix bupleuri*. * *p* < 0.05. ** *p* < 0.01. *** *p* < 0.001. The data shown in the lower left corner were significant at the level of 0.05.

Correlation analysis of 12 trait indicators (including major agronomic and quality traits) of different germplasm of *Radix bupleuri* showed that there are different degrees of correlation among the indicators (Figure 2). Between the agronomic traits of aboveground and underground parts, leaf length was positively correlated with plant height and leaf width, and stem diameter was positively correlated with leaf area. Plant height was significantly and positively correlated with leaf width and leaf area. In contrast, the leaf width/length ratio was negatively correlated with root diameter. After analyzing the correlation between plant indexes and component contents, it was revealed that there was an extremely significant positive correlation between saikosaponin content and root weight. In contrast, leaf width and leaf area were significantly positively correlated with SSd content and total content, and leaf length and root length were positively correlated with total content. SSa content was significantly positively correlated with root length, while negatively correlated with plant height.

### 3.4. Cluster Analysis of Active Components Content

**Figure 3:**
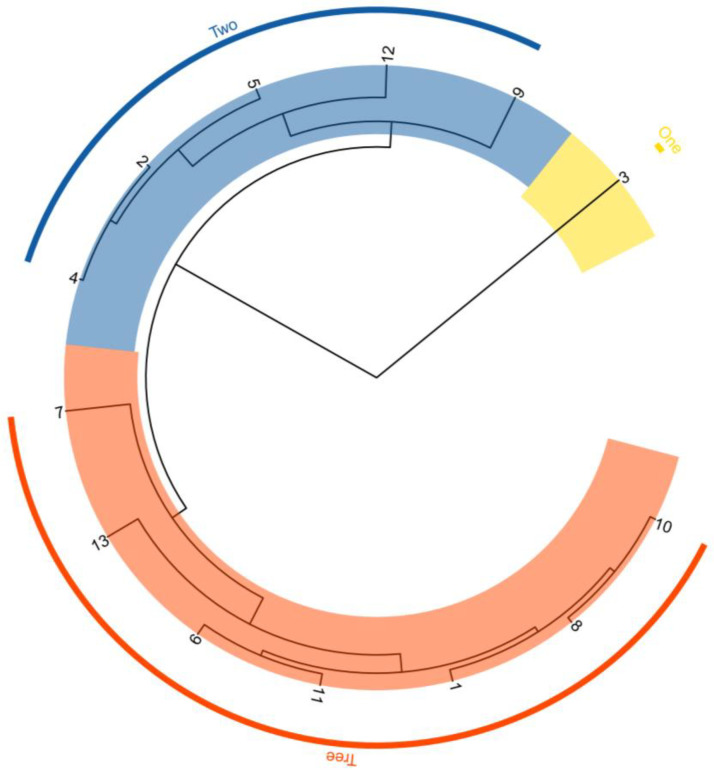
Cluster analysis chart of content traits of different germplasm of *Radix bupleuri*.

Since the quality of *Radix bupleuri* mainly depends on the content of active components in roots, the saponin content of *Radix bupleuri* from different provenances was systematically clustered. The Euclidean distance method was used to calculate the distance between variables, and the average method was used to obtain the clustering tree of 13 *Radix bupleuri* samples (Figure 3). When the similarity coefficient was 6, *Radix bupleuri* samples could be clustered into three classes. Samples No. 2, 4, 5, 9 and 12 were categorized as Class II, samples No. 1, 6, 7, 8, 10, 11 and 13 clustered to Class III, and the germplasm from Shanxi Changzhi was categorized as Class I. It was indicated that the active ingredients varied obviously among different species sources. The medicinal components of *Radix bupleuri* varied significantly from province to province and from different areas of the same province.

### 3.5. ISSR Analysis

**Figure 4:**
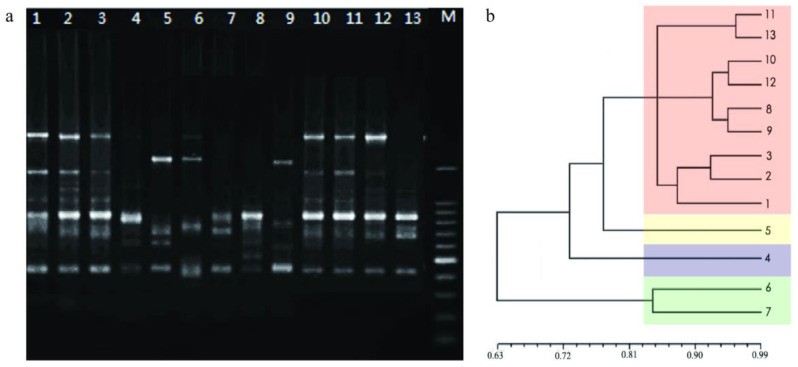
ISSR analysis results. (**a**) ISSR amplification patterns performed by primer 810; (**b**) Dendrogram generated by UPGMA method based on genetic similarity from ISSR marker in *Radix bupleuri* materials.

The nine ISSR primers screened could amplify clear bands from 13 different provenances of *Radix bupleuri* samples (electrophoresis results (810) are shown in Figure 4a). Each pair of primer amplified fragments ranged from 3 to 8. A total of 362 obvious bands were amplified, and polymorphic bands were 325. The proportion of polymorphism was 89.8%, indicating that the selected primers had genetic diversity among 13 individuals, which could be used to detect the genetic diversity of *Radix bupleuri* germplasm resources.

The UPGMA method was used for cluster analysis based on the results of the genetic similarity coefficient, and the results could reflect the genetic relationship among *Radix bupleuri* samples. The similarity coefficient is about 0.78, and 13 *Radix bupleuri* samples from different provenances can be clustered into 4 categories (Figure 4b). The first category included nine *B. chinense* DC. samples, namely Baoji *Radix bupleuri* (Nos. 8–13) and the other three *Radix bupleuri* germplasms from different sources. The second and third categories were *B. smithii* var. *parvifolium* Shan et Y. Li from Lanzhou, Gansu, and *B. falcatum* from Heze, Shandong, respectively. The fourth category was wild samples of *B. marginatum* var. *stenophyllum* (Wolff) Shan et Y. Li. Depending on the germplasm resources, there are apparent differences between *Radix bupleuri* at the molecular level, and their germplasm distinctions can be clearly distinguished using ISSR techniques.

## 4. Discussion

*Radix bupleuri* is one of the major medicinal materials in China. With the research, development, and clinical application of Chinese patent medicines, such as granules, tablets and injections dominated by *Radix bupleuri*, the annual demand for it has surged to 5000 tons, and the limited natural resources are far from meeting the market demand [30]. Currently, the research on *Radix bupleuri* germplasm resources is imperative for breeding improved strains and increasing the supply of high-quality germplasm resources. Due to its variety, extensive range, high phenotypic similarity and ambiguous categorization across species within the genus, it is not easy to identify and utilize the germplasm resources of *Radix bupleuri* [31]. For example, *B. falcatum* is native and growing in Europe and Western Asia. Its main components and pharmacological effects are the same as those of domestic *Radix bupleuri* [15]. It is easy to be confused because of its similarity in shape to other *Radix bupleuri*, especially *B. chinense* DC. *Radix bupleuri* is also mostly sold in the form of processed products (irregular thick slices, vinegar *B. chinense*, turtle blood *B. chinense*), in which the shape and color have changed. This makes it difficult for customers to determine authenticity. Therefore, it is crucial to recognize and safeguard *Radix bupleuri*’s provenance. Numerous morphological, physiological and biochemical studies have already been conducted. However, the identification results of these methods among the more confusing species were far from satisfactory. Han et al. reported that the seeds of *Radix bupleuri* from different habitats have little difference in morphology, which is easily affected by harvesting time and regional environment [32]. Zhang et al. found that the seed phenotypes of *Radix bupleuri* from different varieties showed great differences, as well as the seeds of *B. chinense* DC. from different origins and the same variety, which were similar to the morphological observation results of mature plants [33]. Wang et al. also proved that climatic and geographical factors affected plant growth and secondary metabolism, resulting in regional differences in the active components of *Radix bupleuri* [34]. These results indicated that it was not feasible to judge the variety of *Radix bupleuri* solely by observing the plant appearance and the content of active components.

To eliminate the unpredictable difference between germplasm resources caused by environmental impact, we chose to plant the seeds of *Radix bupleuri* with more varieties in the market in the same field. After studying the agronomic traits and active components, it was discovered that the aboveground part’s coefficient of variation for agronomic traits was 7.62–22.94%, a significant difference from the underground part’s coefficient of variation for agronomic traits, which was 19.01–41.54%. The underground part of *Radix bupleuri* germplasm was where the diversity of the species was most pronounced. Moreover, the Chinese Pharmacopoeia listed SSa and SSd as indicator components for quality control of *Radix bupleuri*, specifying the sum of the two contents ≥0.3% [11]. The content of two saikosaponins in *Radix bupleuri* ranges from 0.730 to 3.123%. All of the materials complied with the requirements, yet there was a substantial difference. Among them, the content of saikosaponin in Changzhi of Shanxi Province was much higher than that in other species of *Radix bupleuri*, which was consistent with the results of cluster analysis based on content. Other different varieties of *Radix bupleuri* fell indiscriminately into the other two categories. Additionally, the close correlation between morphological characteristics and compositional content of the underground parts indicated that environmental factors dominated over genetic impacts in the aboveground components. Morphological characteristics can also reflect a species’ genetic diversity under specific cultivation conditions to some extent. Therefore, plant characters with more notable variation, such as the root traits of *Radix bupleuri*, should receive more focus. For example, the differentiation of commercial specifications and grades based on the size of harvested *Radix bupleuri* is conducive to selecting high-quality medicinal materials. Based on the results of the above studies, it could be seen that even if the heterogeneous *Radix bupleuri* was planted in the same background, differences in appearance and composition would still occur, but such differences could not distinguish the varieties in detail.

As an effective tool for germplasm resource identification and genetic diversity analysis, molecular marker technology has been widely used in genetic background analysis, re-collection of germplasm resources and selection of breeding parents [35]. Many molecular markers have been used to estimate the genetic diversity of *Radix bupleuri* in several investigations. Zhao et al. used the dry roots of cultivated *Radix bupleuri* as the materials and adopted the two analytical methods of RAPD and AFLP to analyze the genetic diversity of *Radix bupleuri,* and they found that the results of the two molecular marker clustering were not completely consistent and could not distinguish *B. falcatum* from *B. chinense* DC [36]. Kang et al. used ITS2 sequences to determine the DNA sequence of the sample seeds. The results of the systematic clustering tree showed that some varieties, such as *B. yinchowense*, were not strictly clustered together. In addition, the clustering of a variety of *Radix bupleuri* plants from different regions further indicated that the ecological environment also had inevitable effects on the genetic variation of *Radix bupleuri* [37]. Song et al. conducted genetic diversity analysis of cultivated and wild *Radix bupleuri* from different regions based on SSR molecular marker technology and found that SSR markers could distinguish the wild population from the cultivated population [38]. However, SSR markers require prior knowledge of the genome, clone or primer design, and the experimental process and data analysis are relatively complex [39].

ISSR is a molecular marker technology with simple operation and high stability. The markers amplify the inter-repeat region. When the plant genome has a high proportion of repeat sequences, it will produce a high polymorphism rate, which can well reveal the polymorphism of species [40]. ISSR has been widely utilized in plant genetic diversity studies. Araújo et al. provided a new approach to determining molecular differences between two cotton species (*Gossypium hirsutum* L. r. marie-galante and *Gossypium barbadense* L.) using the ISSR marker system [41]. Mesfer ALshamrani et al. explored the genetic diversity of six sesame landraces by ISSR maker. They amplified 233 alleles, while the average polymorphism percentage (P%) of all the genotypes studied was 65.32% and the ISSR markers utilized were highly reproducible [42]. Gholami et al. also confirmed that ISSR markers could be useful for distinguishing the relationships among species of Iranian terrestrial orchids [43]. Li et al. analyzed the genetic diversity of 11 samples from 6 populations of *B. chinense* DC. using the ISSR technique and showed that the intraspecific genetic differences of *B. chinense* DC. were related to the environmental differences [44]. Ma and Ke conducted ISSR markers on different varieties of *Radix bupleuri* from different origins but failed to completely distinguish *B. chinense* DC., *B. smithii* Wolff and *B. scorzonerifolium* Willd. It was possible that *Radix bupleuri* was a cross-pollinated plant, and many varieties grew mixed during the cultivation, resulting in germplasm confusion [23]. In addition, the diversity of *Radix bupleuri* based on ISSR molecular marker was rarely reported. In our research, the nine primers reassessed could amplify polymorphic bands, according to our prior laboratory experience, and the UBC 810 primer performed the best. The germplasm materials of No. 4 (*B. falcatum*), No. 5 (*B. smithii* var. *parvifolium* Shan et Y. Li), and *B. chinense* DC. were clustered in different groups, indicating that ISSR markers could accurately and reliably identify *B. chinense* DC. and its adulterants. This approach may prevent errors in judgment caused by the appearance and composition of Chinese herbal medicines. Overall, accurate identification of *Radix bupleuri* germplasm resources are of great significance for the utilization, breeding and production of germplasm and pharmaceutical production with different active ingredients.

## 5. Conclusions

In this study, the morphological characteristics, active component content and the identification of ISSR markers of *Radix bupleuri* from different germplasm resources were comprehensively studied. The results showed that the morphological differences between aboveground parts of different germplasm resources were smaller than those of underground parts, and the morphological characteristics of aboveground parts alone could not completely distinguish the germplasms. The root morphology of different germplasms was significantly correlated with the content of active components, and the saikosaponin contents could be estimated based on the root weight. ISSR technology can accurately distinguish *Radix bupleuri* from different provenances, avoid errors in species identification due to morphology proximity and unclear provenance, screen *Radix bupleuri* counterfeits and prevent medication errors. In addition, the nine polymorphic ISSR primers screened could also be used to detect the genetic diversity of *Radix bupleuri* germplasm resources.

## Figures and Tables

**Table 1:** Specific information of *Radix bupleuri* resources.

No.	Germplasm Source	Breed
1	Datong, Shanxi	*B. chinense* DC.
2	She County, Hebei	*B. chinense* DC.
3	Changzhi, Shanxi	*B. chinense* DC.
4	Heze, Shandong	*B. falcatum*
5	Lanzhou, Gansu	*Bupleurum smithii* var. *parvifolium* Shan et Y. Li
6	Lanzhou, Gansu	*Bupleurum marginatum* var. *stenophyllum* (Wolff) Shan et Y. Li
7	Lanzhou, Gansu	*B. marginatum* var. *stenophyllum* (Wolff) Shan et Y. Li
8	Baoji, Shaanxi	*B. chinense* DC.
9	Baoji, Shaanxi	*B. chinense* DC.
10	Baoji, Shaanxi	*B. chinense* DC.
11	Baoji, Shaanxi	*B. chinense* DC.
12	Baoji, Shaanxi	*B. chinense* DC.
13	Baoji, Shaanxi	*B. chinense* DC.

Note: The variety name was referred to as the Chinese Field Herbarium (CFH: http://www.cfh.ac.cn/default.html (accessed on 17 March 2020)).

**Table 2:** Determination indexes of agronomic traits of *Radix bupleuri*.

No.	Range	Agronomic Trait	Determination Method	Unit
1	Aboveground part	Plant height (PH)	Distance from rhizome bulge to plant apex	cm
2		Stem diameter (SD)	Stem diameter 5 cm upward at base of the main stem	mm
3		Leaf length (LL)	Leaf length of mature middle leaves	cm
4		Leaf width (LW)	Leaf width of mature middle leaves	cm
5		Leaf area (LA)	Leaf area of middle mature leaves	cm^2^
6		Leaf aspect ratio (LAR)	Length–width ratio of middle mature leaves	-
7	Underground part	Root weight (RW)	Weight of clean root	g
8		Root diameter (RD)	Taproot diameter	mm
9		Root length (RL)	Distance from rhizome bulge to the root tip	cm

**Table 3:** Details of 9 primers used in ISSR-PCR.

Number	Primer	Annealing Temperatures (°C)
807	AGAGAGAGAGAGAGAGT	58
809	AGAGAGAGAGAGAGAGG	58
810	GAGAGAGAGAGAGAGAC	52
812	GAGAGAGAGAGAGAGAA	52
823	TCTCTCTCTCTCTCTCC	55
824	GAGAGAGAGAGAGAGAG	55
835	AGAGAGAGAGAGAGAGYC	55
836	AGAGAGAGAGAGAGACYA	55
885	GCCGAGAGAGAGAGAG	52

**Table 4:** Descriptive results of agronomic traits of different *Radix bupleuri* varieties.

No.	PH	SD	LL	LW	LA	LAR	RW	RD	RL
1	102.67 ± 5.51 ^c^	3.42 ± 0.08 ^ab^	8.76 ± 0.66 ^c^	0.84 ± 0.08 ^bc^	6.13 ± 0.78 ^b^	10.40 ± 0.56 ^ab^	1.52 ± 0.28 ^c^	4.77 ± 0.83 ^ab^	19.83 ± 2.75 ^b^
2	123.67 ± 1.53 ^ab^	3.30 ± 0.24 ^ab^	10.07 ± 0.26 ^a–c^	1.07 ± 0.09 ^ab^	8.93 ± 0.98 ^a^	9.16 ± 1.05 ^ab^	1.67 ± 0.16 ^c^	5.37 ± 0.07 ^a^	21.33 ± 2.89 ^b^
3	104.33 ± 5.13^c^	3.48 ± 0.24 ^ab^	-	-	-	-	4.17 ± 0.14 ^a^	3.19 ± 0.21 ^cd^	29.67 ± 1.76 ^a^
4	114.17 ± 6.60 ^a–c^	3.55 ± 0.48 ^ab^	10.77 ± 1.24 ^a–c^	1.10 ± 0.09 ^ab^	9.77 ± 0.36 ^a^	10.89 ± 1.81 ^ab^	-	-	-
5	120.00 ± 7.00 ^ab^	3.81 ± 0.14 ^ab^	10.19 ± 0.88 ^a–c^	1.14 ± 0.15 ^a^	9.66 ± 1.42 ^a^	8.54 ± 0.75 ^b^	1.61 ± 0.28 ^c^	4.33 ± 0.61 ^a-c^	22.67 ± 2.08 ^ab^
6	120.77 ± 0.40 ^ab^	3.41 ± 0.45 ^ab^	10.58 ± 0.57 ^a–c^	1.11 ± 0.07 ^ab^	9.75 ± 0.69 ^a^	8.88 ± 0.42 ^b^	1.33 ± 0.08 ^c^	3.96 ± 0.48 ^b–d^	22.67 ± 4.73 ^ab^
7	114.33 ± 12.01 ^a–c^	3.20 ± 0.07 ^b^	8.61 ± 1.15 ^c^	0.88 ± 0.09 ^a–c^	6.26 ± 1.00 ^b^	10.32 ± 2.13 ^ab^	1.83 ± 0.31 ^c^	4.20 ± 0.27 ^a–c^	23.33 ± 1.53 ^ab^
8	110.67 ± 1.15 ^bc^	3.19 ± 0.18 ^b^	9.13 ± 0.64 ^bc^	0.66 ± 0.02 ^c^	4.97 ± 0.32 ^b^	13.03 ± 1.69 ^a^	1.50 ± 0.26 ^c^	3.68 ± 0.14 ^b–d^	17.33 ± 2.08 ^b^
9	124.17 ± 3.62 ^ab^	3.50 ± 0.38 ^ab^	11.02 ± 1.31 ^a–c^	1.11 ± 0.13 ^ab^	10.23 ± 2.33 ^a^	10.62 ± 0.36 ^ab^	1.95 ± 0.17 ^c^	3.75 ± 0.53 ^b–d^	17.00 ± 2.18 ^b^
10	126.90 ± 2.72 ^a^	3.78 ± 0.14 ^ab^	11.57 ± 0.83 ^ab^	0.99 ± 0.08 ^ab^	9.47 ± 1.02 ^a^	12.18 ± 1.79 ^ab^	1.39 ± 0.28 ^c^	2.90 ± 0.22 ^d^	17.67 ± 2.08 ^b^
11	127.00 ± 2.65 ^a^	3.84±0.16 ^ab^	10.05 ± 1.00 ^a–c^	0.90 ± 0.09 ^a–c^	7.44 ± 0.11 ^ab^	11.26 ± 1.22 ^ab^	2.86 ± 0.73 ^b^	3.69 ± 0.61 ^b–d^	28.67 ± 4.16 ^a^
12	124.67 ± 1.53 ^ab^	4.04±0.29 ^a^	10.77 ± 1.31 ^a–c^	1.06 ± 0.15 ^ab^	9.33 ± 0.44 ^a^	10.41 ± 1.91 ^ab^	2.17 ± 0.35 ^bc^	4.65 ± 0.18 ^ab^	17.17 ± 2.47 ^b^
13	119.83 ± 4.25 ^ab^	3.62±0.28 ^ab^	12.00 ± 0.35 ^a^	1.02 ± 0.08 ^ab^	10.11 ± 0.49 ^a^	11.86 ± 1.18 ^ab^	1.71 ± 0.05 ^c^	3.77 ± 0.49 ^b–d^	20.50 ± 1.32 ^b^

Note: Different letters in the same column showed significant differences (*p* < 0.05).

**Table 5:** Diversity analysis of agronomic traits of different *Radix bupleuri* varieties.

Agronomic Trait	Max	Min	Range	Mean	SD	CV (%)	H’
PH	130.00	97.00	33.00	117.94	8.99	7.62	1.85
SD	4.30	2.89	1.41	3.55	0.34	9.52	1.98
LL	12.53	7.33	5.20	10.29	1.28	12.40	1.99
LW	1.30	0.63	0.67	0.99	0.16	16.44	1.99
LA	12.83	4.65	8.18	8.50	1.95	22.94	1.93
LAR	14.89	7.86	7.03	10.63	1.73	16.31	1.88
RW	4.30	1.07	3.23	1.98	0.82	41.54	1.58
RD	5.45	2.65	2.80	4.02	0.76	19.01	1.98
RL	32.00	14.50	17.50	21.49	4.69	21.83	1.93

**Table 6:** Contents of SSa and SSd in different *Radix bupleuri* varieties.

No.	Content of Components (%)		Total (SST) (%)
	SSa Content (SSA)	SSd Content (SSD)	
1	0.460 ± 0.033 ^b–d^	0.700 ± 0.056 ^cd^	1.160 ± 0.076 ^c–e^
2	0.565 ± 0.087 ^bc^	0.924 ± 0.088 ^bc^	1.489 ± 0.175 ^bc^
3	1.439 ± 0.287 ^a^	1.685 ± 0.213 ^a^	3.123 ± 0.500 ^a^
4	0.579 ± 0.020 ^bc^	0.927 ± 0.040 ^bc^	1.506 ± 0.023 ^bc^
5	0.534 ± 0.015 ^bc^	0.904 ± 0.041 ^bc^	1.438 ± 0.027 ^b–d^
6	0.390 ± 0.039 ^b–d^	0.614 ± 0.037 ^d^	1.004 ± 0.019 ^de^
7	0.263 ± 0.035 ^d^	0.468 ± 0.019 ^d^	0.730 ± 0.053 ^e^
8	0.413 ± 0.053 ^b–d^	0.710 ± 0.087 ^cd^	1.123 ± 0.140 ^c–e^
9	0.337 ± 0.073 ^cd^	1.085 ± 0.039 ^b^	1.421 ± 0.082 ^b–d^
10	0.405 ± 0.029 ^b–d^	0.686 ± 0.027 ^cd^	1.091 ± 0.044 ^c–e^
11	0.431 ± 0.001 ^b–d^	0.641 ± 0.021 ^d^	1.072 ± 0.021 ^c–e^
12	0.637 ± 0.029 ^b^	1.034 ± 0.054 ^b^	1.672 ± 0.077 ^b^
13	0.575 ± 0.073 ^bc^	0.686 ± 0.160 ^cd^	1.261 ± 0.140 ^b–d^

Note: The saikosaponin content was calculate according to the standard curve of the standard substance (SSa: y = 16153x − 315.8, R^2^ = 0.9992; SSd: 18668x − 4525.9, R^2^ = 0.9991). Different letters in the same column showed significant differences (*p* < 0.05).

**Table 7:** Diversity analysis of the content of active ingredients of different *Radix bupleuri* species.

Content Index	Max	Min	Range	Mean	SD	CV (%)	H’
SSa content (SSA)	1.77	0.24	1.53	0.54	0.29	54.18	1.34
SSd content (SSD)	1.93	0.46	1.48	0.85	0.31	36.29	1.66
SSa + SSd content (SST)	3.70	0.69	3.01	1.39	0.58	41.65	1.40

## Data Availability

Not applicable.
